# Supplementary material for: Exploring the strategies and components of interventions to build adolescent awareness about stunting prevention in West Java: A qualitative study
Source: PLoS One. 2024 Dec 5;19(12):e0314651. doi: 10.1371/journal.pone.0314651 (PMC11620421; doi:10.1371/journal.pone.0314651)
Supplement: Attachment — Submitted filename: TS_plos_23082024.pdf [file pone.0314651.s002.pdf]

Thanks to excellent improvement, But to improve the quality paper, please fix some items:

1. Abstract: Clarify the specific objective of exploring interventions, summarize the sub-themes mentioned, and strengthen the practical implications with concrete recommendations for future interventions.
2. Introduction: please provide a sharp gap by explicitly stating the specific gap in existing research or interventions your study aims to address.
3. Conclusion: please strengthen for excellence conclusion by refining the language and emphasizing the significance of the findings.
