# Supplementary material for: Exploring the strategies and components of interventions to build adolescent awareness about stunting prevention in West Java: A qualitative study
Source: PLoS One. 2024 Dec 5;19(12):e0314651. doi: 10.1371/journal.pone.0314651 (PMC11620421; doi:10.1371/journal.pone.0314651)
Supplement: Attachment — Submitted filename: Response to Reviewers.docx [file pone.0314651.s001.docx]

**Authors reply to the comments from the Associate Editor and Reviewers**

**Manuscript ID**	: PONE-D-24-00216

**Tittle**	: Exploring the interventions to build adolescent awareness about

stunting prevention: A qualitative study

Chief of Editor comments

1. Please ensure that your manuscript meets PLOS ONE's style requirements, including those for file naming

**Response:** The authors are most appreciative of the comments from Editor in Chief. We have ensured that our manuscript meets PLOS ONE’s requirements, including those for file naming.

2. Thank you for stating the following financial disclosure:

“This study was supported by PUTI Grant Universitas Indonesia NKB- 336/UN2.RST/HKP.05.00/2023.”

Please state what role the funders took in the study.  If the funders had no role, please state: "The funders had no role in study design, data collection and analysis, decision to publish, or preparation of the manuscript." If this statement is not correct you must amend it as needed. Please include this amended Role of Funder statement in your cover letter; we will change the online submission form on your behalf.

**Response**: The authors thank the editor for the comment. We have added the role of the funder in cover letter as follows:

**Cover letter:**

The funders had no role in study design, data collection and analysis, decision to publish, or preparation of the manuscript.

3. Thank you for stating the following in the Acknowledgments Section of your manuscript:

“The authors would like to thank all participants for their contribution to this study. This study was supported by PUTI Grant Universitas Indonesia NKB- 336/UN2.RST/HKP.05.00/2023.”

We note that you have provided funding information that is currently declared in your Funding Statement. However, funding information should not appear in the Acknowledgments section or other areas of your manuscript. We will only publish funding information present in the Funding Statement section of the online submission form. Please remove any funding-related text from the manuscript and let us know how you would like to update your Funding Statement. Currently, your Funding Statement reads as follows:

“This study was supported by PUTI Grant Universitas Indonesia NKB- 336/UN2.RST/HKP.05.00/2023.”

Please include your amended statements within your cover letter; we will change the online submission form on your behalf.

**Response**: Thank you for the comment. We have removed the funding statement from acknowledgement section to submission form.

4. PLOS requires an ORCID iD for the corresponding author in Editorial Manager on papers submitted after December 6th, 2016. Please ensure that you have an ORCID iD and that it is validated in Editorial Manager. To do this, go to ‘Update my Information’ (in the upper left-hand corner of the main menu), and click on the Fetch/Validate link next to the ORCID field. This will take you to the ORCID site and allow you to create a new iD or authenticate a pre-existing iD in Editorial Manager. Please see the following video for instructions on linking an ORCID iD to your Editorial Manager account: <https://www.youtube.com/watch?v=_xcclfuvtxQ>

**Response**: The author thanks for the suggestion from Editor. We have update the information and validate link next to the ORCID field for each number.

5. Please include your full ethics statement in the ‘Methods’ section of your manuscript file. In your statement, please include the full name of the IRB or ethics committee who approved or waived your study, as well as whether or not you obtained informed written or verbal consent. If consent was waived for your study, please include this information in your statement as well.

**Response**: The authors thank the editor for the suggestion. We have added the information regarding ethics statement including the full name of the IRB and written informed consent in methods section.

**Methods (Page 5, paragraph 1, line 103-106):**

The researcher obtained approval from the nursing faculty's ethics committee at the University of Indonesia. Participants filled out the informed consent form as a sign of agreement to participate in the research after receiving an explanation about the study.

**Reviewer 1**

The study "Exploring the Interventions to Build Adolescent Awareness about Stunting Prevention" used a descriptive qualitative design to investigate interventions for adolescent stunting prevention. this is an important area to touch upon and may bring policy level implication. The feedback on the methodology is as follows:

1. Study Participants:
   The inclusion of high school counselling teachers aimed to gather insights from professionals directly involved with adolescents. The involvement of experts, including doctors, is essential as they possess specialized knowledge relevant to stunting prevention. While including a community nutritionist could provide valuable insights, the specific expertise of the doctors might have been deemed crucial for the study. Considering parents' perspectives could indeed offer valuable insights into the challenges and opportunities for adolescent stunting prevention, and it would be beneficial to include them in future research.

**Response**: We thank authors for the suggestions. We have added the rationale for not including parents in this research in the methods.

**Methods (Page 5, paragraph 2, line 113-116):**

We did not involve parents in the research due to the consideration that if the young generation wants to play an important role in improving their health and become architects of their own future, then the decision-making process must respect the autonomy and choice rights of the young generation (Watson et al., 2023).

2. Online Interviews:
The decision to conduct interviews online might have been influenced by practical considerations such as geographical dispersion of the experts, time efficiency, and cost-effectiveness. However, it's important to acknowledge that online interviews can impact the dynamics of the interaction and the depth of the data collected. Providing a rationale for this choice and addressing any potential implications would strengthen the study.

**Response:** We thank reviewer for the suggestion. We have provided the rationale for conducting interviews online and addressing any potential implication as well as the strategy for preventing those complications in methods.

**Methods (Page 6, paragraph 2, line 132-139)**

Data was gathered through online interviews which considered factors like the geographical spread of experts, time efficiency, and cost savings. Prior to the interviews, researchers looked at challenges with online data gathering such as potential connection issues, ethics guidelines, and ensuring high calibre data (Carter et al., 2021). As such, they aimed to help participants by supplying internet allocations, protecting individual privacy, and only used interviewers who were experienced researchers within the field of childhood stunting and qualitative methods. The preparations sought to address the limitations of remote interviewing and yield meaningful findings.

3. Findings:
Providing a more detailed explanation of the sub-themes within the identified themes, especially the sub-themes of the first theme, could enhance the understanding of the specific insights gained from the participants.

**Response**: The authors thank the reviewer for the suggestion. We have added the detailed explanation of the sub-themes within the identified themes, particularly for the first theme, by providing the table 2. We also provided the information the total corresponds to each sub-themes, and the example of each sub-theme.

**Table 2 (Page 9, line 206-208).**

**Response:**

4. Whole discussion section is missing, please add that as will further strengthen up your paper.
While the study's methodology has several strengths, such as the use of diverse data collection methods and the involvement of relevant participants, addressing the raised points could further enrich the study's comprehensiveness and rigor.

**Response**: We have revised the whole discussion in the manuscript.

Reviewer #2: Thank you for the opportunity to review this manuscript explored the interventions that can be implemented to build adolescents’ awareness about stunting prevention. This study used descriptive qualitative design. The data were collected through focus group discussions and semi-structured interviews to adolescents, high school counselling teachers, and experts. Five themes emerged from this study: 1) Adolescent identity development; 2) Creative and visually appealing website; 3) Nutritional needs for adolescents; 4) Engaging content for adolescents; and 5) Effective communication strategy. Overall the authors explore an important research question, and they used appropriate method to answer the research question. I have a few comments that may help authors improve the quality of the manuscript.

1. The authors reported that they used thematic qualitative analysis. Thematic analysis can either be inductive, deductive, or both. In the way it is written it is not clear if they used deductive, or inductive method.

**Response**: We thank reviewer for the comment. We used inductive and deductive method of thematic analysis. We have explain that information in methods section.

**Methods (Page 7, paragraph 3, line 164-172)**

In this research, we adopted thematic analysis by using deductive and inductive approach. The deductive approach utilizes an organizing framework comprising predetermined themes to systematically code the data (Bradley et al., 2007; Braun and Clarke, 2006; Burnard et al., 2008; Miles and Huberman, 1994). In contrast, the inductive approach involves carefully reading the raw data in detail to derive concepts and themes directly from the content itself without relying on pre-existing constructs. Specifically, this inductive method is implemented by the researcher closely examining each line and paragraph of a participant's statement holistically to reveal the emerging concepts as the text is read. This bottom-up inductive process differs from the top-down nature of the deductive approach and its reliance on an established theoretical structure to guide the analysis.

2. Was there any conceptual framework that guided the research study? If so, which framework was used, and how did the authors analysed data to fit in the domains of the framework? If not, how did the authors develop the themes and sub-themes?

**Response**: We thank reviewer for the comment. We have added the conceptual framework of this research in Conceptual Framework section.

**Conceptual Framework (Page 4, Paragraph 2, line 78-94):**

This conceptual model utilizes Social Learning Theory (SCT) (Bandura, 1977) to prevent stunting among adolescents. It focuses on key concepts from SCT, including observational learning (Bandura, 1977), self-efficacy (Bandura, 1982), behavioral skills (Baranowski et al., 2003), outcome expectations (Bandura, 1986), reinforcement (Bandura, 1986), and social support (Baranowski et al., 2003) to help youth adopt healthy nutrition behaviors. Adolescents will learn positive eating habits by watching nutritionally adept role models in their lives, such as peers without stunting issues (Bandura, 1977).

Nutrition education aims to strengthen self-confidence in managing barriers by building skills such as cooking and meal preparation (Bandura, 1982). Having the ability to perform target behaviors increases the likelihood of taking action (Baranowski et al., 2003). Also, teenagers must believe that positive health outcomes, such as development, will result from their actions (Bandura, 1986). Recognition, such as praise and incentives at home and school, further motivates engagement in nutritious diets (Bandura, 1986). Social learning also occurs through guidance and leading by example from caregivers, educators, and community members who create an encouraging environment conducive to optimal eating (Baranowski et al., 2003). This ensures adequate access to diverse, nutrient-dense foods needed for growth. Addressing these key concepts from social learning theory (Bandura, 1977) empowers adolescents to make informed choices regarding nutrition and prevention of stunting during this important developmental period.

3. It would be great for the authors to describe in the method, what qualitative research guidelines they used (one example is COREQ).

**Response:** We thank reviewer for the comment. We have added the information regarding the guideline in reporting this study on method section.

**Methods (Page 5, paragraph 1, line 101-102):**

We followed the Consolidated Criteria for Reporting Qualitative Research (COREQ) in reporting this study.

4. How many people did code the transcripts, and how were coding conflicts resolved?

**Response**: We have added the information regarding the total number of people did code transcript as well as how were coding conflicts resolved in the methods section.

**Methods (page 7, paragraph 2, line 154-163):**

The initial coding of the transcripts was conducted independently by the first two authors of the study. When the coding list for each set of participants was created, the next step was to identify the similarities and differences in the coding and combined the results from all participants [10]. They then engaged in discussions to determine the final coding framework by combining similar codes and removing duplicate codes. The discussion process yielded a final coding framework that organized the codes into subthemes and main themes aimed at answering the research questions. Additionally, the other authors discussed the potential themes generated at this stage to ensure they fully captured the data obtained through the research process. Any discrepancies that arose during the independent coding were resolved through collaborative discussion between the authors.

5. There are a number of spelling mistakes, I would encourage the authors to proof read the manuscript again. For example, the sentences from line 146 – 148 are similar to 152 – 154.

**Response**: We thank the reviewer for the suggestion. We have proof read the manuscript.

6. In the results, the authors mentioned the themes, sub-themes, and representative quotes. I would recommend the authors use a few sentences to summarize the main message under each theme/ subtheme before stating the quotes. I noted that there are such summaries at the end of each theme, it would be great if these get moved at the beginning, before stating the quotes.

**Response:** We thank reviewer for the suggestions. We have added a few sentences to summarize the main massage under each theme before stating the quotes for each finding in results section.

**Example: Result (Page 11, paragraph 1, line 211-219)**

The participants in the research noted that adolescent identity development needs to be considered in creating educational strategies to avoid stunting in teenagers. Nowadays, young people frequently explore their self-identity online given the simple accessibility of the internet. The participants also stated that the adolescents were in their transition period. As a generation Z, teenagers regularly rebel and rely more on their friends. The power and the role of their peers are crucial. Therefore, taking advantage of the peer group should be taken into account in designing the intervention.
