# Supplementary material for: Exploring the strategies and components of interventions to build adolescent awareness about stunting prevention in West Java: A qualitative study
Source: PLoS One. 2024 Dec 5;19(12):e0314651. doi: 10.1371/journal.pone.0314651 (PMC11620421; doi:10.1371/journal.pone.0314651)
Supplement: Attachment — Submitted filename: Response to Reviewers.docx [file pone.0314651.s003.docx]

**Authors reply to the comments from the Associate Editor and Reviewers**

**Manuscript ID**	: PONE-D-24-00216

**Tittle**	: Exploring the strategies and components of interventions to build

adolescent awareness about stunting prevention in West Java: A

qualitative study

**Chief of Editor comments**

1. Please review your reference list to ensure that it is complete and correct. If you have cited papers that have been retracted, please include the rationale for doing so in the manuscript text, or remove these references and replace them with relevant current references. Any changes to the reference list should be mentioned in the rebuttal letter that accompanies your revised manuscript. If you need to cite a retracted article, indicate the article’s retracted status in the References list and also include a citation and full reference for the retraction notice.

**Response:** The author thanks the editor for the suggestions. We have ensured the references throughout the manuscript, both in citations and the reference list.

**Reviewer 3:**

1. Dear Authors,
   Your article offers valuable insights into building adolescent awareness about stunting prevention. However, several areas need clarification and enhancement to improve its overall quality.

**Response:** The authors truly appreciate the reviewer comments. We will follow the reviewer's suggestions to improve the quality of the manuscript.

2. Tittle: Since Indonesia is so big and having discrepancy on stunting prevalence among provinces, for accuracy of the title, you can consider to add the place study conducted.

**Response:** We thank the reviewer for their suggestions. We have added the place of the study to the title as follows:

**Tittle**

Exploring the strategies and components of interventions to build adolescent awareness about stunting prevention in West Java: A qualitative study

3. Introduction: While the introduction provides a good background on the stunting conceptual framework, it lacks sufficient detail regarding the pivotal factor of teachers, which is also the focus of this research. Consider incorporating primary data, the existing stunting situation in your study place. Providing such information will strengthen the rationale for conducting this research and its contribution to the field of population.

**Response:** The authors thank the reviewer for the comments. We have added the information related to the pivotal factor of teachers in this study and the primary data about prevalence of stunting in the study site in the introduction as follows:

**Introduction**

Stunting is defined as a child's height-for-age being minus two standard deviations (-2SD) from the reference population median [1]. Global projections in 2019 had anticipated the stunting prevalence among children under five years of age would decline, reaching an estimated 21.3% (144 million). However, the prevalence of stunting rose appreciably in Eastern Africa and Asia by approximately 34.5% and 4.5%, respectively [2]. Prior research has shown malnutrition to be directly or indirectly responsible for 30-50% of mortality in children under five years of age, while stunting alone accounts for around 17% of deaths in this paediatric cohort [3]. According to Indonesia's 2018 Basic Health Survey, the national prevalence of stunting was 29.9% for children under two years and 30.8% for those under five [4]. This prevalence declined to 21.6% by 2022. At the subnational level, the prevalence of stunting in the province of West Java fell from 24.5% to 20.2% over this timeframe [5]. However, the decline has yet to reach Indonesia's national target of reducing stunting below 14% by 2024 [6]. Therefore, efforts to address stunting must continue being optimized, with focused interventions targeting demographic subgroups at highest risk.

**Introduction**

School health promotion led by teachers can effectively enhance students' health knowledge and behaviours. Research has shown that educators influence youth development, learning, attitudes, and risks [16]. Teachers are key for health education targeting the development of lifelong healthy practices in students [17]. They also enable open parent-student communication [18]. Active teacher and school involvement thus critically shapes adolescent health understanding. However, lasting behavioural changes require multidimensional prevention coordination across educator and community platforms to optimize consistency in health directives tailored to youth development.

4. Methods: Only 6 adolescent and 5 teacher were interviewed. Can you explain on this. There could have been bias in sampling. We suggest to specify the population size, subject selection methodology, and research process details, including interview conductors and inter-interviewer calibration. Clarify if interviews were individual and if informants were quarantined. Provide information on the interview guide validation or pilot-testing, and mention any limitations if not validated.

**Response:** We thank the reviewer for their suggestions. In conducting the FGDs, we only included 6 adolescents and 5 teachers without specifying the population size. Due to this reason, we have added this point as one of our limitations. Additionally, we have added information about the rationale for involving 5 teachers and 6 students, the inter-interviewer calibration, as well as the interview guide validation or pilot-testing in the methods section.

**Strengths and limitations**

In addition to its strength, this study also has several limitations that should be taken into account. First, the FGDs and interviews were conducted online. It might cause bias because the researchers cannot capture the entire response from the participants. Second, during FGDs with the adolescents, it was difficult to have two-way interaction because some of them seemed shy to explain what they wanted to say. Therefore, in the future, offline FGD and interview are more recommended to make the time and place more conducive for the participants and to gain more optimal data. Third, we only included 5 teachers and 6 adolescents in the FGD. Therefore, there is a possibility of bias in the sample selection. Future research should better consider the population size and conduct subject selection by including participants. Fourth, the study has limited external validity due to its narrow sampling from a single Indonesian province, West Java. Further studies investigating possible variations among subgroups within Indonesia's adolescent population could more robustly reinforce the extent to which these initial results may be extended and applied to other similar contexts nationwide.

**Methods**

Eligibility criteria for the current study included experts, adolescents, and counselling teachers who were organized into three participant groups and interviewed using distinct methods. In-depth interviews were conducted with seven key informants comprising two physicians, two paediatric nurses, one psychologist, and two community nutritionists with expertise in fields relevant to stunting prevention. Concurrently, focus group discussions (FGDs) were held involving five counselling teachers with over two years’ experience guiding adolescents and six secondary-level students. The sample sizes of teacher and student participants in the focus groups were not expanded beyond five and six individuals respectively. This was because additional data and thematic analysis obtained from FGDs conducted separately with adolescent and teacher groups did not uncover any novel themes relating to perceptions of stunting prevention held by these stakeholders. Thematic saturation was achieved within the current purposively selected sample sizes, suggesting further amplification of participant pools would be unlikely to provide extra conceptual insights. The inclusion of 18 total informants was deemed suitable in accordance with established practices in qualitative methodology. No new information emerged upon completing interviews with the 18th participant. Parents were excluded from involvement in the research due to the ethical consideration that empowering young people to play an active role in shaping their own health and futures necessitates respecting the autonomy and self-determination rights of adolescents [27].

**Methods**

In-depth interviews were conducted individually with each participant using a tailored semi-structured interview guide. The interviews were scheduled asynchronously via separate Zoom rooms to maintain confidentiality and prevent response bias. The two interviewers underwent calibration to ensure consistency in administering questions and facilitating techniques. The interview guide was pretested for construct validity and comprehension, with revisions made as needed. This rigorous yet pragmatic methodology aimed to elicit rich qualitative data in an ethical, standardized manner conducive to credible analysis.

5. Discussion: Compare your findings with existing research in Indonesia and note that the study's focus may limit its generalizability to other regions or countries with different cultural dynamics. Addressing these points will make your article more comprehensive, transparent, and informative, thereby improving the overall quality of your research.

**Response:** The authors thank the reviewer for the comment. We have strengthened our manuscript by situating our findings within existing Indonesian research and acknowledging limitations in generalizing due to our regional scope in discussion as follows

**Discussion**

The findings that adolescents spend significant time on social media each day align with past research conducted in Indonesia. Studies by Purboningsih et al. (2022) [36] also found that Indonesian adolescents dedicate more hours to social interaction and browsing online. However, it is important to note that generalizing the exact hours spent online may have limitations depending on regional differences in factors like internet penetration and access to infrastructure within Indonesia. While the influences of digital technologies on enhanced information access and productivity, as highlighted in study of Heidarnia et al. (2016) [37], still hold relevance given the rapid advancement of the digital era, cultural aspects shape adolescents' experiences and behaviours. The study's focus on West Java also means the perspectives may not entirely represent diverse communities across Indonesia's varied geographic, sociocultural, and economic landscapes. Therefore, while digital-based education is appropriate considering participants' screen-based activities, alternative blended approaches could be needed for some rural regions. Additional research exploring potential diversity within the Indonesian adolescent demographic would further strengthen generalization of the findings.

**Discussion**

From the analysis, it is found that the form or variation of the website content needs to be developed by health professionals in designing health education for adolescents. This finding is supported by a study indicating that website-based education is effective in reducing smoking behaviour among adolescents in Kermanshah city, Iran [43] and Indonesia [44]. It seems the aesthetic design with interesting colours and clear layout can enhance visual appeal [45]. In addition, responsive design with smooth animation, appealing images, user-friendliness, and easy navigation can also improve positive interaction among users and increase viewer retention [46, 47]. Developing effective website-based health education for adolescents across Indonesia's diverse regions poses challenges due to significant variations in infrastructure access, connectivity, and digital literacy levels between remote, rural, and more populated areas. Thus, a layered approach tailored to regional contexts is needed to address these challenges and maximize the reach and impact of web-based health interventions for adolescents nationwide.

**Discussion**

The participants' suggestions regarding the use of engaging short videos and interactive content on websites for health education align with past research on optimal multimedia design preferences for Indonesian adolescents. Study by Alfajri et al. (2014) [48] and Ammerlaan et al. (2015) [49] similarly found that adolescents prefer multimedia elements, games, and authentic narratives. However, it is important to note that access to technology and levels of digital literacy can vary substantially between Indonesia's diverse regions and demographics based on urban-rural divides. Cultural norms influencing adolescent help-seeking attitudes also differ nationally, so web-based approaches leveraging peer sharing may need localization [50]. Additionally, capturing the viewpoints of a wider range of adolescent subgroups both geographically and demographically throughout Indonesia could provide further contextualization to strengthen the generalization of implications for optimal website design nationally.

**Reviewer 4**

1. Thanks to excellent improvement, but to improve the quality paper, please fix some items.

**Response:** Thank you for the insightful feedback on how to strengthen our manuscript

2. Abstract: Clarify the specific objective of exploring interventions, summarize the sub-themes mentioned, and strengthen the practical implications with concrete recommendations for future interventions.

**Response:** We thank the reviewer for their comment. We have clarified the specific objective, summarized the sub-themes, and strengthened the practical implications with concrete recommendations for future interventions in the abstract as follows:

**Abstract**

**Aim**: This study aimed to explore the strategies and important components that can be implemented to build adolescent awareness about stunting prevention.

**Methods**: This study used descriptive qualitative design. The data were collected through focus group discussions (FGDs) and semi-structured interviews. Purposive sampling method was employed to select the participants. The FGDs involved adolescents (n=6) and high school counselling teachers (n=5), while the semi-structured interviews were conducted with experts frequently involved in overcoming stunting problems in Indonesia (n=7). The interview results were transcribed in verbatim transcription and analysed by using thematic analysis.

**Results**: Five themes were identified from the results: 1) Adolescent identity development with three sub-themes: online identity exploration, rebellious stage, and peer influence; 2) Creative and visually appealing website with six sub-themes: interesting appearance, short time span, serial content, story pattern, scenario using adolescent idol’s name, and attractive website menu; 3) Nutritional needs for adolescents with three sub-themes: iron and calcium intake, less sugar consumption, and nutritional status; 4) Engaging content for adolescents with seven sub-themes: stunting, reproductive health, anaemia, diet, wellness, early marriage, and physical activity; and 5) Effective communication strategy with two sub-themes: consistency of activities and communicative.

**Implications**: In designing adolescent stunting prevention interventions, multidisciplinary programs utilizing engaging digital health modules and grassroots partnerships should be developed and tested. These programs aim to enhance knowledge retention among youth through appealing online content and interactive community activities. Rigorous evaluation of biopsychosocial approaches can establish integrated best practices across individual, social and policy dimensions for reducing stunting.

3. Introduction: please provide a sharp gap by explicitly stating the specific gap in existing research or interventions your study aims to address.

**Response:** The authors appreciated the reviewer's comment. We have addressed the sharp gap by clearly identifying the specific gap in existing research in the introduction section as follows:

**Introduction**

Stunting education for adolescents has been implemented across Indonesia, including programs such as "PENTINGJADI" in West Sumatra [19], educational media in Central Java [20], and a nutrition curriculum in North Sumatra [21]. These aimed to improve youth nutritional knowledge and attitudes to enable early intervention. However, the development of these platforms did not sufficiently consider adolescents' perspectives on preferred learning methods and topics aligned with their developmental stage. Moreover, few initiatives adopted multidisciplinary collaboration despite stunting's multidimensional nature involving nutrition, health, and education. Addressing this requires cross-disciplinary cooperation throughout program design while meaningfully engaging adolescents and educators. Given adolescents' distinctive needs, tailored accommodation is also needed. This study aims to explore effective strategies and important components that can be implemented to build adolescent awareness about stunting prevention.

4. Conclusion: please strengthen for excellence conclusion by refining the language and emphasizing the significance of the findings

**Response:** We thank the reviewer for their suggestions. We have strengthened the conclusion by emphasizing the significance of our findings and refining the language as follows:

**Conclusions:**

Effective adolescent stunting prevention interventions must consider youth characteristics and preferences identified in this formative study. Findings suggest engaging digital modules delivered through accessible websites, alongside attractive, serialized content of judicious length, have potential to improve nutritional knowledge retention. Curricula addressing identified topics like nutrition, diet and physical activity may boost health behaviours. Consistent, communicative online delivery appears key to sustaining comprehension. This preliminary work informs guidelines for developing tested, tailored programs to holistically prevent stunting among adolescents through improved awareness and involvement in nutritious lifestyle promotion.
